# Potential of Grape Wastes as a Natural Source of Bioactive Compounds

**DOI:** 10.3390/molecules23102598

**Published:** 2018-10-11

**Authors:** Guo-Yi Tang, Cai-Ning Zhao, Qing Liu, Xiao-Ling Feng, Xiao-Yu Xu, Shi-Yu Cao, Xiao Meng, Sha Li, Ren-You Gan, Hua-Bin Li

**Affiliations:** 1 Guangdong Provincial Key Laboratory of Food, Nutrition and Health, Department of Nutrition, School of Public Health, Sun Yat-Sen University, Guangzhou 510080, China; tanggy5@mail2.sysu.edu.cn (G.-Y.T.); zhaocn@mail2.sysu.edu.cn (C.-N.Z.); liuq248@mail2.sysu.edu.cn (Q.L.); xuxy53@mail2.sysu.edu.cn (X.-Y.X.); caoshy3@mail2.sysu.edu.cn (S.-Y.C.); mengx7@mail2.sysu.edu.cn (X.M.); 2 Guangzhou No. 2 High School, Guangzhou 510530, China; 18925022122@163.com; 3 School of Chinese Medicine, Li Ka Shing Faculty of Medicine, The University of Hong Kong, Hong Kong 999077, China; 4 Department of Food Science & Technology, School of Agriculture and Biology, Shanghai Jiao Tong University, Shanghai 200240, China; renyougan@sjtu.edu.cn; 5 South China Sea Bioresource Exploitation and Utilization Collaborative Innovation Center, Sun Yat-Sen University, Guangzhou 510006, China

**Keywords:** grape, peel, seed, waste, antioxidant capacity, bioactive compounds, phenol, flavonoid

## Abstract

Grapes are widely used in the wine and juice industries, which can lead to massive amounts of waste, mostly grape peels and seeds. The antioxidant capacities, total phenolic and flavonoid contents and phenolic profiles of peels and seeds from 30 grape varieties were systemically assessed. The antioxidant activities of fat-soluble, water-soluble and insoluble-bound fractions of grape peels and seeds were evaluated using ferric-reducing antioxidant power and Trolox equivalent antioxidant capacity assays, and their total phenolic contents and total flavonoid contents were determined by the Folin-Ciocalteu method and AlCl_3_ colorimetry, respectively. It was found that the antioxidant capacities were diverse among different grape peels and seeds. Moreover, several phenolic compounds were identified and quantified, including gallic acid, cyanidin-3-glucoside, epicatechin, catechin gallate, ferulaic acid, rutin and resveratrol, which could contribute to the antioxidant capacities of these grape peels and seeds. Several grape wastes with strong antioxidant activity could be abundant sources of natural bioactive compounds, and have the potential for development into functional foods, food additives and pharmaceuticals.

## 1. Introduction

Grape is a famous fruit all over the world, and is widely used in wine and juice industries, which can lead to massive amounts of wastes, including grape peels and seeds [1]. It is reported that these wastes contain a variety of phytochemicals, especially phenols and flavonoids like anthocyanins, resveratrol, tannin and quercetin [2,3,4,5,6,7,8]. These bioactive components possess various outstanding bioactivities, such as antibacterial, anticancer, antioxidant, anti-inflammation and hepatic and cardiovascular protection effects [9,10,11,12,13], and have great safety and effectiveness advantages in preventing chronic diseases [14,15,16,17]. They can be used as raw materials to produce functional foods, food additives and pharmaceuticals [18,19,20,21,22,23]. Many factors can influence the composition and contents of bioactive compounds in fruits, like genotype, growth environment (soil, water, sunlight, etc.) and maturity, among which genotype usually has the greatest impact [24,25,26]. Thus, we could hypothesize that grapes with diverse genotypes should have different composition and contents of bioactive compounds, so it is worthwhile to assess the antioxidant capacities while determining the phenolic and flavonoid contents of peels and seeds from different grape varieties. In the present study, the antioxidant capacities of peels and seeds from 30 grape varieties were measured, and their total phenolic contents and total flavonoid contents were evaluated. In addition, the phenolic and flavonoid constituents were identified and quantified using HPLC analysis. This should prove helpful for the full utilization of grape peels and seeds.

## 2. Results and Discussion

### 2.1. Ferric Reducing Antioxidant Power (FRAP) of the Grape Peels and Seeds

The FRAP was used as one of the indices to assess antioxidant capacities of these grape peels and seeds. The FRAP assay is established on the basis of the ability that antioxidants reduce ferric ions to ferrous ions [27], which is a simple and commonly employed method to evaluate antioxidant capacity [28,29,30]. The FRAP values of these grape peels and seeds are presented in Table 1.

For the 30 grape peels, the total FRAP values varied from 18.304 ± 0.680 to 252.983 ± 9.185 μmol Fe(II)/g fresh weight (FW) with a 14-fold difference. Blackcurrant Grape (California, CA, USA), Golden Finger Grape (California, CA, USA), Seedless Black Grape (California, CA, USA), Summer Black Grape (Shaanxi, China) and Black Grape (Yunnan, China) possessed the top-five antioxidant capacities, which were 252.983 ± 9.185, 222.155 ± 5.954, 197.742 ± 11.638, 157.761 ± 10.846 and 153.706 ± 5.904 μmol Fe(II)/g FW, respectively. Fragrant Green Grape (Yunnan, China) possessed the lowest antioxidant capacity, which was 18.304 ± 0.680 μmol Fe(II)/g FW. In addition, the ranges of FRAP values for three fractions were in a decreasing order: fat-soluble (6.734 ± 0.364 to 161.671 ± 5.628 μmol Fe(II)/g FW) > water-soluble (11.407 ± 0.311 to 115.195 ± 0.595 μmol Fe(II)/g FW) > insoluble-bound (0.074 ± 0.005 to 0.614 ± 0.032 μmol Fe(II)/g FW) (*p* = 0.030, *p* < 0.001, respectively).

For the 10 grape seeds, the total FRAP values varied from 312.429 ± 11.760 to 858.121 ± 35.507 μmol Fe(II)/g FW with a 3-fold difference. Pearl Black Grape (Xinjiang, China), Black Grape (Yunnan, China), Red Grape (Yunnan, China), Red Grape (Xinjiang, China) and Red Grape (California, CA, USA) possessed the top-five antioxidant capacities, which were 858.121 ± 35.507, 837.242 ± 21.578, 616.485 ± 29.629, 562.018 ± 19.437 and 520.390 ± 19.974, respectively. Kyoho Grape (Guangxi, China) possessed the lowest antioxidant capacity, which was 312.429 ± 11.760 μmol Fe(II)/g FW. In addition, the ranges of FRAP values for three fractions were in a decreasing order: fat-soluble (250.876 ± 8.208 to 726.495 ± 23.487 μmol Fe(II)/g FW) > water-soluble (44.148 ± 3.996 to 144.767 ± 3.348 μmol Fe(II)/g FW) > insoluble-bound (0.383 ± 0.033 to 1.881 ± 0.184 μmol Fe(II)/g FW) (*p* < 0.001, *p* = 0.038, respectively).

According to the results described above, the FRAP values of fat-soluble fractions were generally higher than those of water-soluble fractions, which were distinctly higher than those of insoluble-bound fractions. These results indicated that the antioxidants responsible for the reducing power of grape peels and seeds were most fat-soluble compounds with some water-soluble and a little insoluble-bound ones. When evaluating total antioxidant capacities of grape peels and seeds, all of the three fractions should be taken into consideration. In addition, the FRAP values of grape seeds were apparently higher than those of grape peels (*p* < 0.001). As compared to other materials, the FRAP values of the tested grape peels were higher than those of most edible macro-fungi, vegetables, fruits and fruit wastes (peels and seeds) [31,32,33,34], and also higher than those of some wild fruits and edible and wild flowers [35,36]. Moreover, the FRAP values of the tested grape seeds were higher than those of most edible macro-fungi, vegetables, wild fruits, edible and wild flowers, fruits and fruit wastes (peels and seeds) [31,32,33,34,35,36], and higher than those of some medicinal plants [37]. So grape peels and seeds could be abundant resources of natural antioxidants with great potential to produce functional foods, food additives and pharmaceuticals.

### 2.2. Trolox Equivalent Antioxidant Capacity (TEAC) of the Grape Peels and Seeds

Most natural antioxidants are multifunctional, and the antioxidant capacities of plant samples are generally impacted by multiple factors, such as the extraction solvent, extraction method and measurement method, leading to difficulty to completely demonstrate antioxidant capacities using a single method. Therefore, an authentic antioxidant assessing system requires evaluations of multiple aspects, and it is essential to conduct different experiments to assess the antioxidant activity which might be associated with diverse mechanisms of action [38]. The TEAC assay is a simple, fast, repeatable and widely used method for the evaluation of antioxidant capacity [39,40]. The TEAC assay is on the basis of the capability of antioxidants to scavenge the ABTS^•+^ radical, and can be used for measuring antioxidant capacities of fat-soluble, water-soluble and insoluble-bound components in the same sample [41]. As reported, vitamin C, vitamin E, butylated hydroxytoluene, butylated hydroxyanisole and Trolox were often applied as reference standards [42,43]. Here, Trolox was employed. The TEAC values of the peels and seeds from 30 grape varieties are displayed in Table 2.

For the 30 grape peels, the total TEAC values ranged from 5.176 ± 0.209 to 123.740 ± 2.969 μmol Trolox/g FW with a 24-fold difference. Blackcurrant Grape (California, CA, USA), Seedless Black Grape (Xinjiang, China), Golden Finger Grape (California, CA, USA), Summer Black Grape (Shaanxi, China) and Black Grape (Yunnan, China) possessed the top-five free radical scavenging capacities, which were 123.740 ± 2.969, 101.151 ± 3.839, 98.106 ± 5.902, 82.242 ± 4.086 and 82.053 ± 2.861 μmol Trolox/g FW, respectively. Fragrant Green Grape (Yunnan, China) possessed the lowest free radical scavenging capacity, which was 5.176 ± 0.209 μmol Trolox/g FW. In addition, the ranges of TEAC values for three fractions were in a decreasing order: fat-soluble (2.293 ± 0.133 to 84.463 ± 1.361 μmol trolox/g FW) > water-soluble (2.726 ± 0.061 to 39.110 ± 1.592 μmol Trolox/g FW) > insoluble-bound (0.028 ± 0.002 to 0.383 ± 0.029 μmol trolox/g FW) (*p* < 0.001, *p* < 0.001, respectively).

For the 10 grape seeds, the total TEAC values ranged from 207.815 ± 10.573 to 473.454 ± 19.303 μmol Trolox/g FW with a 2-fold difference. Pearl Black Grape (Xinjiang, China), Black Grape (Yunnan, China), Red Grape (Yunnan, China), Red Grape (Xinjiang, China) and Red Grape (California, CA, USA) possessed the top-five free radical scavenging capacities, which were 473.454 ± 19.303, 392.577 ± 6.236, 330.155 ± 13.086, 293.910 ± 8.804 and 292.349 ± 8.610 μmol Trolox/g FW, respectively. Kyoho Grape (Yunnan, China) possessed the lowest free radical scavenging capacity, which was 207.815 ± 10.573 μmol Trolox/g FW. In addition, the ranges of TEAC values for three fractions were in a decreasing order: fat-soluble (181.739 ± 1.029 to 409.190 ± 19.195 μmol trolox/g FW) > water-soluble (24.555 ± 1.907 to 64.105 ± 0.106 μmol trolox/g FW) > insoluble-bound (0.159 ± 0.003 to 1.320 ± 0.071 μmol trolox/g FW) (*p* < 0.001, *p* = 0.023, respectively).

As seen from the description before, the TEAC values of fat-soluble fractions were generally higher than those of water-soluble fractions, which were distinctly higher than those of insoluble-bound fractions. It meant that the antioxidants, which were responsible for the free radical scavenging activities of grape peels and seeds, were most fat-soluble compounds with some water soluble and a little insoluble-bound ones. When the total antioxidant capacities of grape peels and seeds are about to be assessed, three fractions should all be counted in. In addition, the TEAC values of the grape seeds were extremely higher than those of the grape peels (*p* < 0.001). Besides, the TEAC values of the grape peels were higher than those of most edible macro-fungi, vegetables, fruits and fruit waste (peels and seeds) [31,32,33,34], and higher than those of some wild fruits and edible and wild flowers [35,36]. Furthermore, the TEAC values of the grape seeds were higher than those of most edible macro-fungi, vegetables, edible and wild flowers, fruits and fruit waste (peels and seeds) [31,32,33,34,35,36], and higher than those of some wild fruits and medicinal plants [37], so grape peels and seeds could be developed into functional foods, food additives and pharmaceuticals regarding antioxidants.

### 2.3. Total Phenolic Contents (TPC) of 30 Grape Peels and 10 Grape Seeds

The TPC values of these grape peels and seeds were determined by the Folin-Ciocalteu method, which is based on the reaction that electrons are transferred from phenolic compounds to the Folin-Ciocalteu reagent in alkaline medium, and is a simple, rapid and reproducible method [44]. The TPC values of these grape peels and seeds are given in Table 3.

For the 30 grape peels, the total TPC values varied from 1.588 ± 0.062 to 25.724 ± 0.894 mg GAE/g FW with a 16-fold difference. Blackcurrant Grape (California, CA, USA), Seedless Black Grape (Xinjiang, China), Kyoho Grape (Liaoning, China), Pearl Black Grape (Xinjiang, China) and Summer Black Grape (Shaanxi, China) had the top-five total phenolic contents, which were 25.724 ± 0.894, 20.153 ± 0.983, 15.483 ± 1.006, 15.338 ± 0.897 and 14.822 ± 0.879 mg GAE/g FW, respectively. Fragrant Green Grape (Yunnan, China) had the lowest total phenolic content, which was 1.588 ± 0.062 mg GAE/g FW. In addition, the ranges of TPC values for three fractions were in a decreasing order: fat-soluble (0.811 ± 0.025 to 16.528 ± 0.463 mg GAE/g FW) > water-soluble (0.754 ± 0.037 to 9.1705 ± 0.4299 mg GAE/g FW) > insoluble-bound (0.011 ± 0.001 to 0.072 ± 0.002 mg GAE/g FW) (*p* = 0.009, *p* < 0.001, respectively).

For the 10 grape seeds, the total TPC values varied from 34.628 ± 2.435 to 71.244 ± 0.762 mg GAE/g FW with a 2-fold difference. Pearl Black Grape (Xinjiang, China), Black Grape (Yunnan, China), Red Grape (Yunnan, China), Red Grape (Xinjiang, China) and Red Grape (California, CA, USA) had the top-five total phenolic contents, which were 71.244 ± 0.762, 70.376 ± 1.207, 55.771 ± 1.912, 51.315 ± 1.578 and 49.170 ± 1.570 mg GAE/g FW, respectively. Kyoho Grape (Yunnan, China) had the lowest total phenolic content, which was 34.628 ± 2.435 mg GAE/g FW. In addition, the ranges of TPC values for three fractions were in a decreasing order: fat-soluble (28.584 ± 2.017 to 58.372 ± 0.692 mg GAE/g FW) > water-soluble (5.593 ± 0.365 to 13.150 ± 0.249 mg GAE/g FW) > insoluble-bound (0.039 ± 0.001 to 0.214 ± 0.009 mg GAE/g FW) (*p* < 0.01, *p* = 0.02, respectively).

Based on the above demonstration, the TPC values of the grape seeds were drastically higher than those of the grape peels (*p* < 0.001). Besides, the TPC values of the grape peels were higher than those of most edible macro-fungi, vegetables, fruits and fruit waste (peels and seeds) [31,32,33,34], and higher than those of some wild fruits and edible and wild flowers [35,36]. Moreover, the TPC values of the grape seeds were higher than those of most edible macro-fungi, vegetables, edible and wild flowers, wild fruits, fruits and fruit wastes (peels and seeds) [31,32,33,34,35,36], and higher than those of some medicinal plants [37], so grape peels and seeds could be used to extract phenols with further applications in the functional food, pharmaceutical, food additive and cosmetic industries. Furthermore, it should be pointed out that some non-phenolic components such as organic acids and sugars, which also possess reducing capacity, could affect the measurement of total phenolic contents determined by the Folin–Ciocalteu method, leading to overestimated total phenolic contents [45,46]. In addition, varied phenols might response to the Folin–Ciocalteu reagent differently and several flavonoids present low responses, which might cause an underestimate of total phenolic contents [47,48,49].

### 2.4. Total Flavonoid Contents (TFC) of the Grape Peels and Seeds

The TFC values of these grape peels and seeds were estimated by the AlCl_3_ colorimetry method according to the literature reported by Kalia et al., which is based on the reaction that the 3-hydroxy-4-hydroxyl or 5-hydroxy-4-carbonyl or *o*-2-phenolic hydroxyl of flavonoids is combined with Al^3+^ to form a red complex under an alkaline condition, and is a simple, rapid and repeatable method [50]. The TFC values of these grape peels and seeds are given in Table 4.

For the 30 grape peels, the total TFC values ranged from 0.176 ± 0.005 to 1.408 ± 0.091 mg QE/g FW with an 8-fold difference. Blackcurrant Grape (California, CA, USA), Golden Finger Grape (California, CA, USA), Seedless Black Grape (Xinjiang, China), Black Grape (Yunnan, China) and Rose Black Grape (Xinjiang, China) had the top-five total flavonoid contents, which were 1.408 ± 0.091, 1.130 ± 0.084, 0.982 ± 0.056, 0.962 ± 0.031 and 0.649 ± 0.027 mg QE/g FW, respectively. Pearl Green Grape (Xinjiang, China) had the lowest total flavonoid content, which was 0.176 ± 0.005 mg QE/g FW. In addition, the ranges of TPC values for three fractions were in a decreasing order: fat-soluble (0.109 ± 0.002 to 1.017 ± 0.087 mg QE/g FW) > water-soluble (0.042 ± 0.001 to 0.381 ± 0.003 mg QE/g FW) > insoluble-bound (0.008 ± 0.000 mg QE/g FW to 0.081 ± 0.004 mg QE/g FW) (*p* < 0.001, *p* = 0.001, respectively).

For the 10 grape seeds, the total TFC values ranged from 1.130 ± 0.054 mg QE/g FW to 3.957 ± 0.213 mg QE/g FW with a 4-fold difference. Red Grape (Yunnan, China), Kyoho Grape (Xinjiang, China), Pearl Black Grape (Xinjiang, China), Ito Kyoho Grape(Yunnan, China) and Kyoho Grape (Guangxi, China) had the top-five total flavonoid contents, which were 3.957 ± 0.213, 3.884 ± 0.189, 3.626 ± 0.176, 3.122 ± 0.022 and 2.765 ± 0.245 mg QE/g FW, respectively. Red Grape (California, CA, USA) had the lowest total flavonoid content, which was 1.130 ± 0.054 mg QE/g FW. In addition, the ranges of TPC values for three fractions were in a decreasing order: fat-soluble (1.024 ± 0.044 to 3.792 ± 0.211 mg QE/g FW) > water-soluble (0.074 ± 0.006 mg QE/g FW to 0.173 ± 0.003 mg QE/g FW) > insoluble-bound (0.020 ± 0.001 to 0.146 ± 0.006 mg QE/g FW) (*p* < 0.001, *p* = 0.815, respectively).

As illustrated before, the TFC values of the grape seeds were higher than those of the grape peels (*p* < 0.001). Both of the TFC values of the grape peels and seeds were lower than those of most medicinal plants and some common plant/tree waste [51,52]. Moreover, extracts with higher TPC values did not always have higher TFC values, different extracts contained different levels of TFC as a portion of phenols [51,52,53]. So it should be pointed out that grape peels and seeds were valuable resources of natural phenols but not flavonoids.

### 2.5. Correlations between Total FRAP, TEAC, TPC and TFC Values

**Figure 1:**
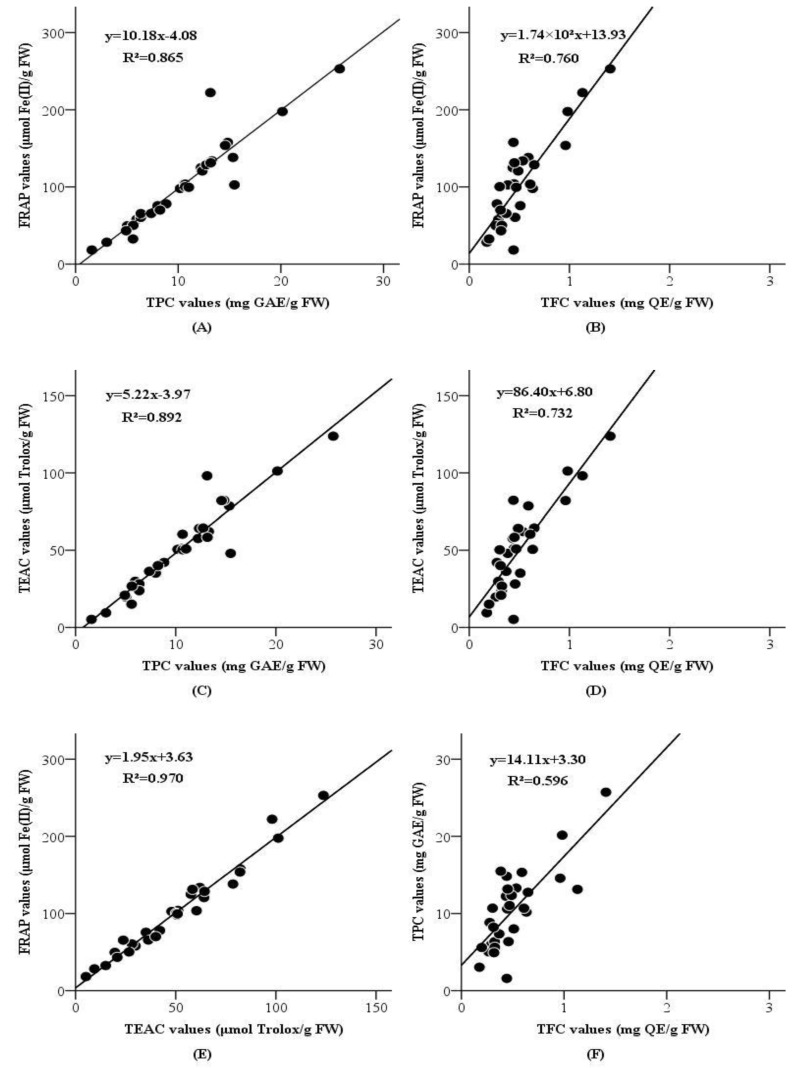
Correlations between FRAP values and TPC values (**A**); FRAP values and TFC values (**B**); TEAC values and TPC values (**C**); TEAC values and TFC values (**D**); FRAP values and TEAC values (**E**); TPC values and TFC values (**F**) of peels from 30 grape varieties.

**Figure 2:**
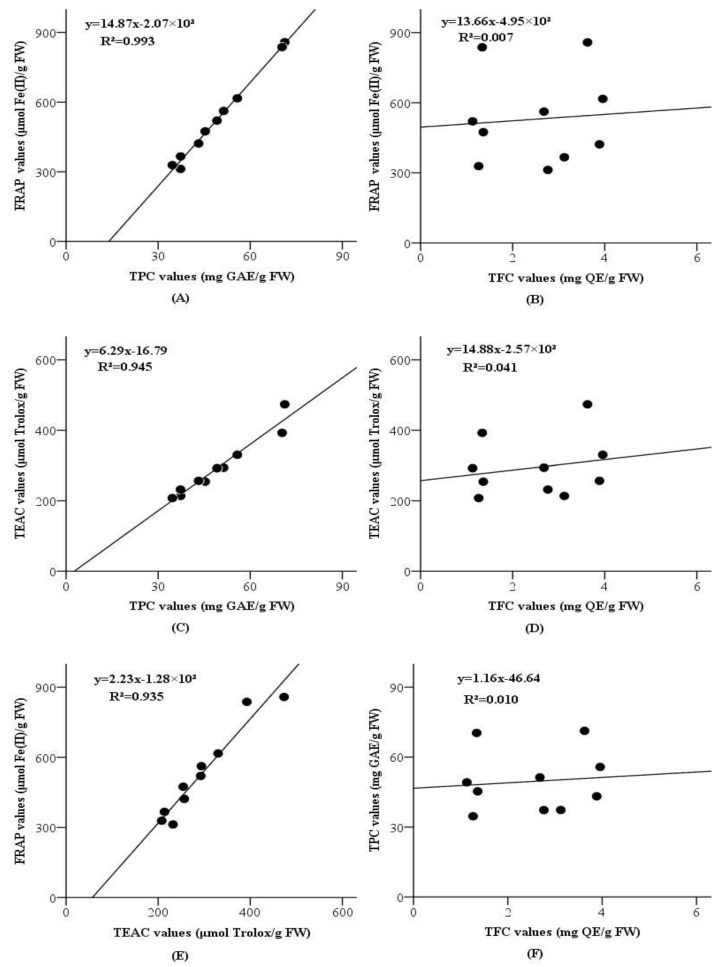
Correlations between FRAP values and TPC values (**A**); FRAP values and TFC values (**B**); TEAC values and TPC values (**C**); TEAC values and TFC values (**D**); FRAP values and TEAC values (**E**); TPC values and TFC values (**F**) of seeds collected from 10 grape varieties.

The correlations between FRAP, TEAC, TPC and TFC values (based on the total values of three fractions) were detected using a simple linear regression model, and the results were displayed in Figure 1 and Figure 2.

For grape peels, as seen from Figure 1, FRAP values and TEAC values were highly correlated to TPC values (R² = 0.865, *p* < 0.001 and R² = 0.892, *p* < 0.001, respectively), and moderately correlated to TFC values (R² = 0.760, *p* < 0.001 and R² = 0.732, *p* < 0.001, respectively). The outcomes revealed that phenolic components could be the main ingredients responsible for the antioxidant capacities of the grape peels, and flavonoid compounds might contribute to the antioxidant capacities of grape peels but were not the main contributors. In addition, TPC values were weakly correlated with TFC values (R² = 0.596, *p* < 0.001). It suggested that flavonoids comprised only a small part of phenolic components of the grape peels. Furthermore, FRAP values were significantly correlated with TEAC values (R² = 0.970, *p* < 0.001), so the antioxidant ingredients in the grape peels could reduce oxidants (like Fe(III)) and scavenge free radicals (like ABTS^•+^).

For grape seeds, according to Figure 2, FRAP values and TEAC values were intensely correlated to TPC values (R² = 0.993, *p* < 0.001 and R² = 0.945, *p* < 0.001, respectively), but not correlated to TFC values (R² = 0.007, *p* = 0.825 and R² = 0.041, *p* = 0.574, respectively). The outcomes suggested that phenolic components could be the main contributors to the antioxidant capacities of the grape seeds, but flavonoid compounds had little influence on the antioxidant capacities of grape seeds. Additionally, there was no linear correlation between TPC values and TFC values (R² = 0.010, *p* = 0.779), which suggested that phenolic components of the grape seeds were rarely flavonoids. Moreover, the correlation between FRAP values and TEAC values was remarkable (R² = 0.935, *p* < 0.001), so the antioxidant components in these grape seeds could also reduce oxidants (like Fe(III)) and scavenge free radicals (like ABTS^•+^).

The results illustrated above are consistent with many previous studies, which have reported that phenolic components were the main contributors responsible for the antioxidant capacities, and could reduce oxidants and scavenge free radicals [31,33,34,35,36]. On the contrary, these results were quite different from some other studies that reported a very weak correlation (R^2^ = 0.0337) between the FRAP values and TEAC values [32,37], indicating that the ingredients possessing reducing activities and those possessing free radicals scavenging activities in the 62 fruits were not the same, and a very weak correlation (R^2^ = 0.0404) between the TEAC values and TPC values, suggesting that phenolic components could not be the main contributors to the free radicals scavenging abilities of the 62 fruits. Li et al. [37] also reported a very weak correlation between the TEAC values and the FRAP values (R^2^ = 0.1563) as well as the FRAP values and the TPC values (R^2^ = 0.1966), which suggested that phenolic components could not be the main contributors to activities of the 223 medicinal plants to reduce oxidants.

### 2.6. Phenolic Components of the Grape Peels and Seeds

**Figure 3:**
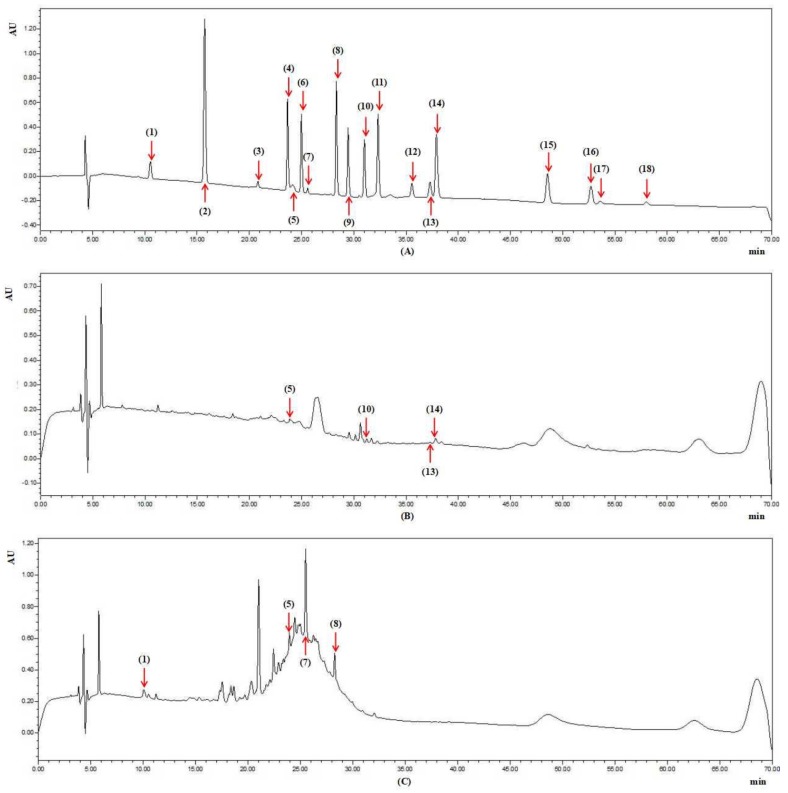
Chromatograms under 220 nm of the standard compounds (**A**); Black Grape (Yunnan, China) peel (**B**); Pearl Black Grape (Xinjiang, China) seed (**C**).The numbers in brackets refer to the compounds: gallic acid (**1**); protocatechuic acid (**2**); gallo catechin (**3**); chlorogenic acid (**4**); cyanidin-3-glucoside (**5**); caffeic acid (**6**); epicatechin (**7**); catechin gallate (**8**); *p*-coumaric acid (**9**); ferulaic acid (**10**); melatonin (**11**); 2-hydroxycinnamic acid (**12**); rutin (**13**); resveratrol (**14**); daidzein (**15**); equol (**16**); quercetin (**17**); genistein (**18**).

Phenolic components of the grape peels and seeds were determined on the base of the literature reported by Cai et al. with small alteration [54]. Phenolic components of the grape peels and seeds were detected, and the results were displayed in Table 5. Furthermore, the chromatograms under 220 nm of the mixed standards and the samples of Black Grape (Yunnan, China) peel and Pearl Black Grape (Xinjiang, China) seed were shown in Figure 3.

As seen from Table 5, five phenols, including cyanidin-3-glucoside, epicatechin, rutin, ferulaic acid and resveratrol, were found in the 30 grape peels. Every grape peel sample contained rutin, and the contents ranged from 0.008 ± 0.000 to 0.804 ± 0.055 mg/g FW with a 100-fold difference. The peel of Red Grape (Yunnan, China) possessed the highest level of rutin. Some grape peels contained cyanidin-3-glucoside, and the contents ranged from 0.021 ± 0.001 to 0.498 ± 0.028 mg/g FW with a 24-fold difference. The peel of Blackcurrant Grape (California, CA, USA) possessed the highest level of cyanidin-3-glucoside. The peels of Summer Black Grape (Shaanxi, China), Kyoho Grape (Xinjiang, China) and Ito Kyoho Grape (Yunnan, China) contained epicatechin of 0.051 ± 0.004, 0.026 ± 0.002 and 0.015 ± 0.001 mg/g FW, and the peels of Black Grape (Yunnan, China), Flame Grape (Xinjiang, China) and Golden Finger Grape (California, CA, USA) contained ferulaic acid of 0.241 ± 0.011, 0.049 ± 0.003 and 0.041 ± 0.003 mg/g FW, while resveratrol (0.266 ± 0.015 mg/g FW) was only detected in the peel of Black Grape (Yunnan, China).

As for the 10 grape seeds, four phenols including gallic acid, cyanidin-3-glucoside, epicatechin and catechin gallate were found in all of them, and the content ranges were as follows, respectively: 0.022 ± 0.001 to 0.236 ± 0.009 mg/g FW with a 10-fold difference; 0.058 ± 0.003 to 0.840 ± 0.052 mg/g FW with a 14-fold difference; 0.877 ± 0.065 to 2.156 ± 0.156 mg/g FW with a 2-fold difference; 0.028 ± 0.002 to 0.176 ± 0.008 mg/g FW with a 7-fold difference, respectively. The seeds of Red Grape (Yunnan, China), Ito Kyoho Grape (Yunnan, China), Red Grape (Yunnan, China) and Red Grape (Yunnan, China) possessed the highest level of gallic acid, cyanidin-3-glucoside, epicatechin and catechin gallate, respectively. These results also prove our hypothesis that grapes with diverse genotypes have different composition and contents of bioactive compounds. Furthermore, it was reported that phenols like cyanidin-3-glucoside, resveratrol and rutin possessed varies bioactivities, such as antibacterium, antioxidant, anti-inflammation and hepatic and cardiovascular protection, so grape peels and seeds from juice and wine industries could be valuable resources to extract phenols with further use in producing functional foods, food additives and pharmaceuticals.

The above results were expressed on the weight of fresh material. In addition, the moisture contents of the grape peels and seeds are displayed in Table 6, which could be used to express the results on the weight of dry material.

## 3. Materials and Methods

### 3.1. Chemical Reagents

The 2,2′-azinobis(3-ethylbenothiazoline-6-sulphonic acid) diammonium salt (ABTS), 6-hydroxy-2,5,7,8-tetramethylchromane-2-carboxylic acid (Trolox), 2,4,6-tri(2-pyridyl)-*s*-triazine (TPTZ), Folin-Ciocalteu’s phenol reagent, and the standard compounds (gallic acid, protocatechuic acid, gallo catechin, chlorogenic acid, cyanidin-3-glucoside, caffeic acid, epicatechin, catechin gallate, *p*-coumaric acid, ferulaic acid, melatonin, 2-hydroxycinnamic acid, rutin, resveratrol, daidzein, equol, quercetin and genistein) were provided by Sigma-Aldrich (St. Louis, MO, USA). Tetrahydrofuran, methanol, formic acid, diethyl ether and ethyl acetate were provided by Kermel Chemical Factory (Tianjin, China). Acetic acid, sodium acetate, potassium acetate, sodium hydroxide, hydrochloric acid, ethylenediaminetetraacetic acid (EDTA), ascorbic acid, iron (III) chloride hexahydrate (FeCl_3_·6H_2_O), iron(II) sulphate heptahydrate (FeSO_4_·7H_2_O), potassium persulphate, sodium carbonate, aluminum chloride hexahydrate (AlCl_3_·6H_2_O), ethanol and *n*-hexane were provided by Damao Chemical Factory (Tianjin, China). All chemical reagents used in the tests were of analytical or chromatographic grade, and the water used was double distilled.

### 3.2. Sample Preparation

**Figure 4:**
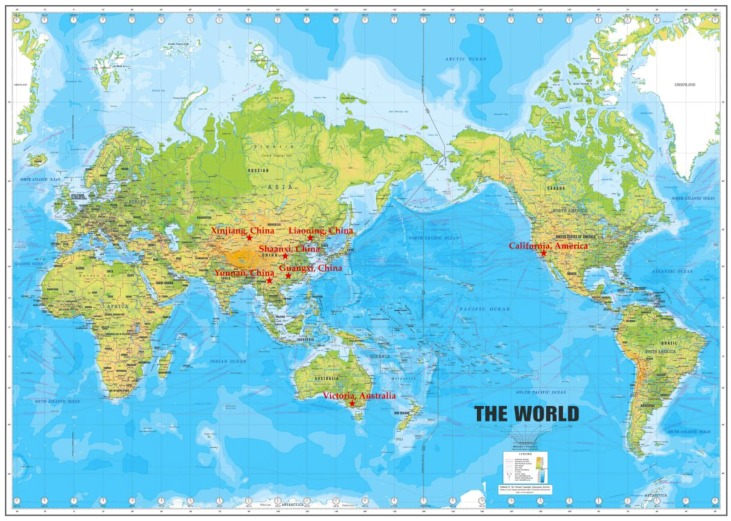
Coordinates of geographical areas of the tested grapes.

Grapes from 30 varieties produced in China, USA and Australia (Figure 4) were obtained from local shops in Guangzhou, China. Grapes were washed with double distilled water and dried at room temperature. The grapes were separated into peels, pulps and seeds, and then the peels and seeds were respectively ground into particles using a special grinder for food processing. After that, accurate 2.000 g of the samples were weighed and extracted with 10 mL tetrahydrofuran at 30 °C for 30 min in a shaking water bath [55]. The samples were centrifuged at 4200 g for 10 min, and the supernatants were gathered. The extraction was repeated twice, and the supernatants were collected as the fat-soluble fractions. Subsequently, the residues were extracted with 10 mL methanol-acetic acid-water (50:3.7:46.3, *v/v/v*) mixture at 30 °C for 30 min in a shaking water bath, which was also repeated twice, and the supernatants were gathered up as the water-soluble fractions. Furthermore, the residues were hydrolyzed with 5 mL sodium hydroxide solution (2 mol/L NaOH, 10 mmol/L EDTA, 1% ascorbic acid) at 37 °C for 30 min in a shaking water bath, and then acidified to pH = 2 with 6 mol/L hydrochloric acid solution [56]. The mixtures were extracted twice with 5 mL *n*-hexane to eliminate fatty acids, which might be released during alkaline hydrolysis. Immediately, the mixtures were extracted twice with 5 mL diethyl ether and ethyl acetate mixture (1:1, *v/v*), and the organic phases were collected. The extracts were dried out at room temperature under a stream of N_2_ using an evaporator and reconstituted in ethanol as the insoluble-bound fractions. All extracts were preserved at −20 °C until tested.

### 3.3. FRAP Assay

The FRAP assay was conducted referring to the literature with minor alterations [27]. Briefly, the FRAP reagent was a mixture of 300 mmol/L sodium acetate-acetic acid buffer (pH = 3.6), 10 mmol/L TPTZ solution (40 mmol/L hydrochloric acid solution as solvent) and 20 mmol/L FeCl_3_ solution (10:1:1, *v/v/v*), and it was prepared freshly and warmed to 37 °C in a water bath before used. The 0.1 mL properly diluted sample was combined with 3 mL FRAP reagent. After incubated at room temperature for 4 min, a CANY 722 visible spectrophotometer (Shanghai, China) was used to measure the absorbance of the mixtures at 593 nm. The size and volume of cuvette were 1 cm × 1 cm × 4.5 cm and 4.5 mL, respectively. The assay volumes were 1/2 to 2/3 of the volume of cuvette. The results were expressed as μmol Fe (II)/g FW of the grape peels or seeds.

### 3.4. TEAC Assay

The TEAC assay was carried out according to the literature with minor alterations [41]. Accordingly, the ABTS^•+^ stock solution was a mixture of 7 mmol/L ABTS solution and 2.45 mmol/L potassium persulphate solution (1:1, *v/v*), which was incubated in the dark for at least 16 h at room temperature and used within 2 days. The ABTS^•+^ working solution was obtained by diluting the stock solution with ethanol to an absorbance of 0.710 ± 0.05 at 734 nm. The samples were diluted approximately until they can inhibit 20–80% blank absorbance. Subsequently, the 0.1 mL properly diluted sample was mixed with 3.8 mL ABTS^•+^ working solution and measured at 734 nm after incubated at room temperature for 6 min. The percent of inhibition of absorbance was calculated to evaluate of the antioxidant capacity. The results were expressed as μmol Trolox/g FW of the grape peels or seeds.

### 3.5. Determination of TPC

The TPC values were determined based on procedures reported by Singleton, Orthofer and Lamuela-Raventos [49]. Briefly, a properly diluted sample (0.5 mL) was added to Folin-Ciocalteu reagent (0.2 mol/L, 2.5 mL). After 4 min, saturated sodium carbonate solution (about 75 g/L, 2 mL) was added to the mixture. The mixture was incubated at room temperature for 2 h, and then the absorbance was measured at 760 nm. The results were expressed as milligram gallic acid equivalent (mg GAE)/g FW of the grape peels or seeds.

### 3.6. Determination of TFC

The TFC values were determined according to the literature reported by Kalia et al. [50]. Accordingly, a properly diluted sample (0.5 mL) was mixed with ethanol solution (95%, *v/v*, 1.5 mL), AlCl_3_ solution (10%, *w/v*, 0.1 mL), potassium acetate solution (1 mol/L, 0.1 mL) and double distilled water (2.8 mL). The mixture was incubated for 30 min at room temperature, and then the absorbance was measured at 415 nm. The results were expressed as mg quercetin equivalent (mg QE)/g FW of the grape peels or seeds.

### 3.7. HPLC Analysis

The phenolic and flavonoid components in the samples were detected by HPLC-PDAD (photodiode array detector) based on the method reported by Cai et al. with small modifcations [54]. In detail, the HPLC system included a Waters (Milford, MA, USA) 1525 binary HPLC pump separation module with an auto-injector and employed a Waters 2996 PDAD. Separation was carried out with an Agilent Zorbax Extend-C18 column (250 × 4.6 mm, 5 μm) at 40 °C with a gradient elution solution A, composed of formic acid solution (0.1%, *v/v*), and solution B, methanol, which were routinely delivered at a flow rate of 0.8 mL/min according to the procedure: 0 min, 95% (A); 15 min, 80% (A); 20 min, 70% (A); 25 min, 63% (A); 40 min, 60% (A); 60 min, 50% (A); 65 min, 50% (A); 65.1 min, 95% (A); and 70 min, 95% (A). Fat-soluble, water-soluble and insoluble-bound fractions were combined together before sampling. The spectra were recorded between 200 and 600 nm to characterize the peak patterns. Phenolic and flavonoid components were identified by the retention time and UV-Vis spectra comparing with standards and quantified by the peak area under maximum absorption wavelength, and the results were expressed as mg/g FW of the grape peels or seeds.

### 3.8. Data Analysis

All tests were conducted in triplicate and the values were expressed as mean ± SD (standard deviation). Data analysis was performed using SPSS 22 (International Business Machines Corporation, Armonk, NY, USA) and Excel 2007 (Microsoft Corporation, Redmond, WA, USA).

## 4. Conclusions

In this study, the antioxidant capacities and total phenolic and flavonoid contents of peels and seeds from 30 grape varieties were systematically evaluated. The antioxidant capacities and phenolic and flavonoid contents of the grape peels and seeds were greatly different, and those of the three fractions were, in decreasing order: fat-soluble fractions > water-soluble fractions > insoluble-bound fractions. Antioxidant components in these grape peels and seeds could reduce oxidants and scavenge free radicals, and phenols were the main contributors to the antioxidant capacities, and flavonoids were not major contributors to these activities. Several phenolic compounds such as gallic acid, cyanidin-3-glucoside, epicatechin, catechin gallate, ferulaic acid, rutin and resveratrol were identified and quantified in these grape peels and seeds. These grape wastes could be abundant sources of natural bioactive compounds for developing functional foods, food additives and pharmaceuticals.

## Figures and Tables

**Table 1:** FRAP values of peels and seeds from 30 grape varieties.

Name of Grapes	Place of Production	Part of Grapes	FARAP Values (μmol Fe(II)/g FW)			
			Fat-Soluble Fraction	Water-Soluble Fraction	Insoluble-Bound Fraction	Total
Black Grape	Yunnan, China	Peel	99.407 ± 4.048	54.026 ± 1.833	0.273 ± 0.024	153.706 ± 5.904
Blackcurrant Grape	California, CA, USA	Peel	161.671 ± 5.628	91.100 ± 3.554	0.211 ± 0.003	252.983 ± 9.185
Flame Grape	Xinjiang, China	Peel	24.241 ± 2.288	40.931 ± 1.694	0.286 ± 0.022	65.457 ± 4.004
ragrant Green Grape	Yunnan, China	Peel	6.734 ± 0.364	11.407 ± 0.311	0.163 ± 0.005	18.304 ± 0.680
Golden Finger Grape	California, CA, USA	Peel	106.886 ± 5.354	115.195 ± 0.595	0.074 ± 0.005	222.155 ± 5.954
Green Grape	Victoria, Australia	Peel	20.336 ± 0.398	22.645 ± 1.450	0.245 ± 0.010	43.226 ± 1.858
Ito Kyoho Grape	Yunnan, China	Peel	63.526 ± 4.318	40.336 ± 1.193	0.170 ± 0.015	104.032 ± 5.526
Kyoho Grape	Guangxi, China	Peel	94.002 ± 2.110	26.026 ± 2.239	0.614 ± 0.032	120.642 ± 4.380
Kyoho Grape	Liaoning, China	Peel	59.621 ± 1.689	15.907 ± 0.655	0.164 ± 0.007	75.693 ± 2.351
Kyoho Grape	Xinjiang, China	Peel	90.217 ± 5.724	12.407 ± 1.111	0.092 ± 0.005	102.716 ± 6.840
Kyoho Grape	Yunnan, China	Peel	75.812 ± 5.004	24.264 ± 2.020	0.254 ± 0.023	100.331 ± 7.047
Pearl Black Grape	Xinjiang, China	Peel	84.479 ± 2.465	53.600 ± 3.536	0.144 ± 0.004	138.223 ± 6.005
Pearl Green Grape	Xinjiang, China	Peel	7.288 ± 0.648	20.907 ± 0.842	0.154 ± 0.013	28.350 ± 1.503
Pearl Green Grape	Victoria, Australia	Peel	36.955 ± 1.041	40.883 ± 1.636	0.211 ± 0.013	78.050 ± 2.689
Red Grape	California, CA, USA	Peel	42.169 ± 3.351	27.741 ± 0.975	0.083 ± 0.003	69.992 ± 4.329
Red Grape	Guangxi, China	Peel	35.645 ± 1.221	24.764 ± 0.446	0.131 ± 0.011	60.541 ± 1.678
Red Grape	Xinjiang, China	Peel	43.812 ± 1.782	21.693 ± 1.823	0.133 ± 0.013	65.638 ± 3.617
Red Grape	Yunnan, China	Peel	62.336 ± 2.133	35.431 ± 2.093	0.203 ± 0.005	97.969 ± 4.231
Rose Black Grape	Xinjiang, China	Peel	62.526 ± 3.635	65.717 ± 5.437	0.387 ± 0.030	128.630 ± 9.103
Rose Black Grape	Yunnan, China	Peel	59.383 ± 2.580	39.741 ± 1.034	0.408 ± 0.033	99.532 ± 3.646
Seedless Black Grape	California, CA, USA	Peel	106.674 ± 2.619	90.669 ± 8.983	0.401 ± 0.036	197.742 ± 11.638
Seedless Black Grape	Xinjiang, China	Peel	66.526 ± 2.269	67.074 ± 3.610	0.219 ± 0.003	133.819 ± 5.882
Seedless Dew Grape	Xinjiang, China	Peel	18.241 ± 1.656	31.136 ± 1.262	0.295 ± 0.015	49.672 ± 2.933
Seedless Green Grape	Xinjiang, China	Peel	7.264 ± 0.707	25.169 ± 1.567	0.131 ± 0.010	32.564 ± 2.284
Seedless Red Grape	California, CA, USA	Peel	53.657 ± 0.051	71.217 ± 4.113	0.094 ± 0.003	124.967 ± 4.166
Seedless Red Grape	Victoria, Australia	Peel	65.669 ± 2.402	65.288 ± 5.794	0.294 ± 0.003	131.251 ± 8.198
Seedless Red Grape	Xinjiang, China	Peel	24.026 ± 0.664	26.002 ± 1.329	0.138 ± 0.005	50.167 ± 1.998
Seedless Red Grape	Yunnan, China	Peel	25.669 ± 0.972	31.979 ± 1.608	0.217 ± 0.005	57.864 ± 2.586
Summer Black Grape	Shaanxi, China	Peel	85.121 ± 6.061	72.407 ± 4.772	0.233 ± 0.013	157.761 ± 10.846
Summer Black Grape	Xinjiang, China	Peel	56.883 ± 5.017	46.312 ± 3.526	0.379 ± 0.012	103.574 ± 8.555
Black Grape	Yunnan, China	Seed	692.019 ± 18.217	144.767 ± 3.348	0.456 ± 0.013	837.242 ± 21.578
Ito Kyoho Grape	Yunnan, China	Seed	301.924 ± 4.439	64.148 ± 4.014	0.424 ± 0.000	366.495 ± 8.452
Kyoho Grape	Xinjiang, China	Seed	371.448 ± 13.718	50.243 ± 3.295	0.436 ± 0.023	422.127 ± 17.036
Kyoho Grape	Guangxi, China	Seed	250.876 ± 8.208	59.671 ± 3.369	1.881 ± 0.184	312.429 ± 11.760
Kyoho Grape	Yunnan, China	Seed	283.638 ± 22.325	44.148 ± 3.996	0.868 ± 0.052	328.654 ± 26.373
Pearl Black Grape	Xinjiang, China	Seed	726.495 ± 23.487	131.243 ± 11.987	0.383 ± 0.033	858.121 ± 35.507
Red Grape	Yunnan, China	Seed	502.876 ± 27.668	112.862 ± 1.918	0.747 ± 0.043	616.485 ± 29.629
Red Grape	Guangxi, China	Seed	351.067 ± 19.144	122.576 ± 3.677	0.528 ± 0.005	474.170 ± 22.825
Red Grape	Xinjiang, China	Seed	450.305 ± 15.144	111.243 ± 4.267	0.470 ± 0.027	562.018 ± 19.437
Red Grape	California, CA, USA	Seed	401.924 ± 16.337	117.148 ± 3.570	1.319 ± 0.068	520.390 ± 19.974

**Table 2:** TEAC values of peels and seeds from 30 grape varieties.

Name of Grapes	Place of Production	Part of Grapes	TEAC Values (μmol Trolox/g FW)			
			Fat-Soluble Fraction	Water-Soluble Fraction	Insoluble-Bound Fraction	Total
Black Grape	Yunnan, China	Peel	58.264 ± 2.194	23.4742 ± 0.637	0.315 ± 0.030	82.053 ± 2.861
Blackcurrant Grape	California, CA, USA	Peel	84.463 ± 1.361	39.1097 ± 1.59	0.167 ± 0.015	123.740 ± 2.969
Flame Grape	Xinjiang, China	Peel	14.572 ± 1.001	9.0073 ± 0.140	0.209 ± 0.016	23.788 ± 1.157
Fragrant Green Grape	Yunnan, China	Peel	2.293 ± 0.133	2.7259 ± 0.061	0.156 ± 0.015	5.1760 ± 0.209
Golden Finger Grape	California, CA, USA	Peel	68.596 ± 5.519	29.4711 ± 0.381	0.039 ± 0.003	98.106 ± 5.902
Green Grape	Victoria, Australia	Peel	13.165 ± 0.524	7.4739 ± 0.314	0.166 ± 0.016	20.804 ± 0.854
Ito Kyoho Grape	Yunnan, China	Peel	34.777 ± 2.078	16.1091 ± 0.714	0.242 ± 0.023	51.128 ± 2.815
Kyoho Grape	Guangxi, China	Peel	31.151 ± 1.088	3.8408 ± 0.206	0.138 ± 0.003	35.130 ± 1.297
Kyoho Grape	Liaoning, China	Peel	42.555 ± 0.447	5.3211 ± 0.081	0.069 ± 0.003	47.945 ± 0.531
Kyoho Grape	Xinjiang, China	Peel	50.852 ± 3.082	12.8820 ± 0.979	0.383 ± 0.029	64.117 ± 4.090
Kyoho Grape	Yunnan, China	Peel	38.092 ± 1.912	12.0422 ± 1.146	0.163 ± 0.008	50.297 ± 3.065
Pearl Black Grape	Xinjiang, China	Peel	48.202 ± 0.567	30.2866 ± 2.763	0.075 ± 0.005	78.563 ± 3.336
Pearl Green Grape	Xinjiang, China	Peel	3.267 ± 0.254	6.0273 ± 0.026	0.141 ± 0.013	9.435 ± 0.293
Pearl Green Grape	Victoria, Australia	Peel	21.066 ± 0.750	20.9262 ± 0.525	0.105 ± 0.010	42.097 ± 1.285
Red Grape	California, CA, USA	Peel	34.654 ± 0.605	15.7459 ± 0.266	0.158 ± 0.010	50.557 ± 0.881
Red Grape	Guangxi, China	Peel	21.460 ± 0.485	6.5821 ± 0.043	0.098 ± 0.008	28.141 ± 0.536
Red Grape	Xinjiang, China	Peel	26.268 ± 2.316	9.8911 ± 0.620	0.134 ± 0.011	36.293 ± 2.947
Red Grape	Yunnan, China	Peel	24.813 ± 1.079	15.1921 ± 0.525	0.028 ± 0.002	40.033 ± 1.606
Rose Black Grape	Xinjiang, China	Peel	35.038 ± 1.647	29.0777 ± 2.282	0.237 ± 0.016	64.353 ± 3.944
Rose Black Grape	Yunnan, China	Peel	30.936 ± 1.431	19.7177 ± 0.469	0.216 ± 0.009	50.869 ± 1.909
Seedless Black Grape	California, CA, USA	Peel	34.388 ± 0.258	27.5440 ± 1.616	0.116 ± 0.008	62.047 ± 1.882
Seedless Black Grape	Xinjiang, China	Peel	62.835 ± 0.877	38.0697 ± 2.950	0.246 ± 0.012	101.151 ± 3.839
Seedless Dew Grape	Xinjiang, China	Peel	11.461 ± 0.428	7.9079 ± 0.043	0.252 ± 0.003	19.621 ± 0.473
Seedless Green Grape	Xinjiang, China	Peel	4.579 ± 0.363	10.3781 ± 0.402	0.083 ± 0.008	15.040 ± 0.773
Seedless Red Grape	California, CA, USA	Peel	16.143 ± 0.473	13.5667 ± 0.159	0.150 ± 0.010	29.860 ± 0.642
Seedless Red Grape	Victoria, Australia	Peel	14.900 ± 0.758	11.7903 ± 0.731	0.033 ± 0.003	26.724 ± 1.491
Seedless Red Grape	Xinjiang, China	Peel	29.336 ± 1.267	28.2014 ± 1.974	0.044 ± 0.003	57.582 ± 3.243
Seedless Red Grape	Yunnan, China	Peel	36.584 ± 1.474	21.6865 ± 1.676	0.103 ± 0.005	58.374 ± 3.155
Summer Black Grape	Shaanxi, China	Peel	51.789 ± 1.878	30.2866 ± 2.195	0.167 ± 0.013	82.242 ± 4.086
Summer Black Grape	Xinjiang, China	Peel	31.496 ± 2.283	28.6149 ± 1.900	0.246 ± 0.009	60.358 ± 4.192
Black Grape	Yunnan, China	Seed	329.773 ± 5.710	62.5184 ± 0.510	0.287 ± 0.015	392.577 ± 6.236
Ito Kyoho Grape	Yunnan, China	Seed	181.739 ± 1.029	31.7674 ± 1.296	0.295 ± 0.023	213.802 ± 2.348
Kyoho Grape	Xinjiang, China	Seed	231.921 ± 9.528	24.5545 ± 1.907	0.207 ± 0.005	256.682 ± 11.440
Kyoho Grape	Guangxi, China	Seed	192.411 ± 9.735	38.4204 ± 1.567	1.320 ± 0.071	232.152 ± 11.373
Kyoho Grape	Yunnan, China	Seed	176.024 ± 8.804	31.2584 ± 1.739	0.532 ± 0.031	207.815 ± 10.573
Pearl Black Grape	Xinjiang, China	Seed	409.190 ± 19.195	64.1050 ± 0.106	0.159 ± 0.003	473.454 ± 19.303
Red Grape	Yunnan, China	Seed	274.455 ± 11.707	55.1126 ± 1.364	0.587 ± 0.015	330.155 ± 13.086
Red Grape	Guangxi, China	Seed	199.613 ± 6.407	54.3559 ± 1.308	0.380 ± 0.005	254.349 ± 7.720
Red Grape	Xinjiang, China	Seed	246.250 ± 7.908	47.3937 ± 0.889	0.266 ± 0.007	293.910 ± 8.804
Red Grape	California, CA, USA	Seed	229.045 ± 7.867	62.7832 ± 0.696	0.520 ± 0.048	292.349 ± 8.610

**Table 3:** TPC values of peels and seeds from 30 grape varieties.

Name of Grapes	Place of Production	Part of Grapes	TPC Values (mg GAE/g FW)			
			Fat-Soluble Fraction	Water-Soluble Fraction	Insoluble-Bound Fraction	Total
Black Grape	Yunnan, China	Peel	8.992 ± 0.646	5.516 ± 0.091	0.066 ± 0.006	14.574 ± 0.742
Blackcurrant Grape	California, CA, USA	Peel	16.529 ± 0.463	9.171 ± 0.430	0.025 ± 0.001	25.724 ± 0.894
Flame Grape	Xinjiang, China	Peel	2.5485 ± 0.173	3.782 ± 0.144	0.029 ± 0.002	6.356 ± 0.319
Fragrant Green Grape	Yunnan, China	Peel	0.811 ± 0.025	0.754 ± 0.037	0.024 ± 0.001	1.588 ± 0.062
Golden Finger Grape	California, CA, USA	Peel	6.443 ± 0.488	6.673 ± 0.024	0.015 ± 0.001	13.131 ± 0.514
Green Grape	Victoria, Australia	Peel	2.530 ± 0.028	2.366 ± 0.087	0.029 ± 0.002	4.925 ± 0.118
Ito Kyoho Grape	Yunnan, China	Peel	6.778 ± 0.300	3.774 ± 0.139	0.047 ± 0.004	10.599 ± 0.444
Kyoho Grape	Guangxi, China	Peel	6.809 ± 0.176	1.177 ± 0.061	0.018 ± 0.001	8.003 ± 0.239
Kyoho Grape	Liaoning, China	Peel	8.719 ± 0.341	6.752 ± 0.664	0.012 ± 0.001	15.483 ± 1.006
Kyoho Grape	Xinjiang, China	Peel	9.341 ± 0.517	2.935 ± 0.095	0.072 ± 0.002	12.348 ± 0.614
Kyoho Grape	Yunnan, China	Peel	7.689 ± 0.713	2.962 ± 0.151	0.036 ± 0.003	10.687 ± 0.866
Pearl Black Grape	Xinjiang, China	Peel	8.374 ± 0.210	6.944 ± 0.686	0.020 ± 0.001	15.338 ± 0.897
Pearl Green Grape	Xinjiang, China	Peel	1.138 ± 0.014	1.872 ± 0.083	0.027 ± 0.002	3.037 ± 0.099
Pearl Green Grape	Victoria, Australia	Peel	4.189 ± 0.089	4.618 ± 0.148	0.027 ± 0.002	8.833 ± 0.239
Red Grape	California, CA, USA	Peel	6.624 ± 0.233	3.528 ± 0.178	0.037 ± 0.001	10.189 ± 0.412
Red Grape	Guangxi, China	Peel	4.006 ± 0.400	2.331 ± 0.175	0.022 ± 0.001	6.359 ± 0.575
Red Grape	Xinjiang, China	Peel	4.935 ± 0.242	2.398 ± 0.043	0.029 ± 0.001	7.362 ± 0.286
Red Grape	Yunnan, China	Peel	4.683 ± 0.325	3.532 ± 0.084	0.011 ± 0.001	8.226 ± 0.410
Rose Black Grape	Xinjiang, China	Peel	6.524 ± 0.295	6.171 ± 0.486	0.043 ± 0.004	12.738 ± 0.786
Rose Black Grape	Yunnan, China	Peel	6.319 ± 0.560	4.669 ± 0.086	0.045 ± 0.002	11.032 ± 0.648
Seedless Black Grape	California, CA, USA	Peel	6.758 ± 0.193	6.512 ± 0.345	0.023 ± 0.000	13.293 ± 0.538
Seedless Black Grape	Xinjiang, China	Peel	11.426 ± 0.278	8.679 ± 0.703	0.048 ± 0.002	20.153 ± 0.983
Seedless Dew Grape	Xinjiang, China	Peel	2.372 ± 0.146	2.626 ± 0.185	0.041 ± 0.001	5.039 ± 0.332
Seedless Green Grape	Xinjiang, China	Peel	2.795 ± 0.026	2.774 ± 0.099	0.025 ± 0.002	5.594 ± 0.126
Seedless Red Grape	California, CA, USA	Peel	2.992 ± 0.052	2.933 ± 0.041	0.028 ± 0.002	5.953 ± 0.095
Seedless Red Grape	Victoria, Australia	Peel	2.839 ± 0.144	2.764 ± 0.113	0.016 ± 0.000	5.619 ± 0.257
Seedless Red Grape	Xinjiang, China	Peel	5.667 ± 0.086	6.553 ± 0.308	0.012 ± 0.001	12.232 ± 0.395
Seedless Red Grape	Yunnan, China	Peel	6.599 ± 0.110	6.547 ± 0.611	0.023 ± 0.002	13.169 ± 0.723
Summer Black Grape	Shaanxi, China	Peel	8.624 ± 0.535	6.169 ± 0.342	0.029 ± 0.002	14.822 ± 0.879
Summer Black Grape	Xinjiang, China	Peel	5.606 ± 0.078	5.036 ± 0.318	0.043 ± 0.001	10.685 ± 0.397
Black Grape	Yunnan, China	Seed	57.169 ± 0.954	13.150 ± 0.249	0.057 ± 0.004	70.376 ± 1.207
Ito Kyoho Grape	Yunnan, China	Seed	29.966 ± 0.098	7.300 ± 0.403	0.045 ± 0.002	37.311 ± 0.503
Kyoho Grape	Xinjiang, China	Seed	37.527 ± 0.483	5.593 ± 0.365	0.054 ± 0.005	43.174 ± 0.853
Kyoho Grape	Guangxi, China	Seed	29.998 ± 1.621	7.073 ± 0.139	0.214 ± 0.009	37.285 ± 1.769
Kyoho Grape	Yunnan, China	Seed	28.584 ± 2.017	5.947 ± 0.411	0.098 ± 0.006	34.628 ± 2.435
Pearl Black Grape	Xinjiang, China	Seed	58.372 ± 0.692	12.833 ± 0.069	0.039 ± 0.001	71.244 ± 0.762
Red Grape	Yunnan, China	Seed	44.714 ± 1.636	10.967 ± 0.269	0.090 ± 0.008	55.771 ± 1.912
Red Grape	Guangxi, China	Seed	33.917 ± 1.436	11.333 ± 0.279	0.062 ± 0.006	45.312 ± 1.722
Red Grape	Xinjiang, China	Seed	40.811 ± 1.199	10.451 ± 0.374	0.053 ± 0.005	51.315 ± 1.578
Red Grape	California, CA, USA	Seed	37.104 ± 1.315	11.971 ± 0.253	0.095 ± 0.002	49.170 ± 1.570

**Table 4:** TFC values of peels and seeds from 30 grape varieties.

Name of Grapes	Place of Production	Part of Grapes	TFC Values (mg QE/g FW)			
			Fat-Soluble Fraction	Water-Soluble Fraction	Insoluble-Bound Fraction	Total
Black Grape	Yunnan, China	Peel	0.688 ± 0.021	0.260 ± 0.009	0.014 ± 0.001	0.962 ± 0.031
Blackcurrant Grape	California, CA, USA	Peel	1.017 ± 0.087	0.381 ± 0.003	0.010 ± 0.000	1.408 ± 0.091
Flame Grape	Xinjiang, China	Peel	0.175 ± 0.011	0.139 ± 0.005	0.010 ± 0.000	0.324 ± 0.016
Fragrant Green Grape	Yunnan, China	Peel	0.320 ± 0.027	0.042 ± 0.001	0.081 ± 0.004	0.443 ± 0.032
Golden Finger Grape	California, CA, USA	Peel	0.760 ± 0.059	0.362 ± 0.025	0.008 ± 0.000	1.130 ± 0.084
Green Grape	Victoria, Australia	Peel	0.232 ± 0.011	0.075 ± 0.002	0.010 ± 0.001	0.318 ± 0.014
Ito Kyoho Grape	Yunnan, China	Peel	0.302 ± 0.025	0.090 ± 0.005	0.055 ± 0.002	0.448 ± 0.032
Kyoho Grape	Guangxi, China	Peel	0.425 ± 0.017	0.072 ± 0.002	0.013 ± 0.001	0.510 ± 0.020
Kyoho Grape	Liaoning, China	Peel	0.326 ± 0.022	0.049 ± 0.003	0.008 ± 0.000	0.384 ± 0.026
Kyoho Grape	Xinjiang, China	Peel	0.402 ± 0.023	0.071 ± 0.006	0.016 ± 0.001	0.488 ± 0.029
Kyoho Grape	Yunnan, China	Peel	0.229 ± 0.023	0.063 ± 0.005	0.012 ± 0.001	0.304 ± 0.029
Pearl Black Grape	Xinjiang, China	Peel	0.398 ± 0.020	0.133 ± 0.011	0.059 ± 0.001	0.590 ± 0.032
Pearl Green Grape	Xinjiang, China	Peel	0.109 ± 0.002	0.052 ± 0.002	0.015 ± 0.001	0.176 ± 0.005
Pearl Green Grape	Victoria, Australia	Peel	0.192 ± 0.002	0.074 ± 0.002	0.011 ± 0.000	0.276 ± 0.004
Red Grape	California, CA, USA	Peel	0.504 ± 0.023	0.115 ± 0.006	0.013 ± 0.001	0.633 ± 0.029
Red Grape	Guangxi, China	Peel	0.343 ± 0.019	0.104 ± 0.005	0.011 ± 0.000	0.458 ± 0.024
Red Grape	Xinjiang, China	Peel	0.283 ± 0.018	0.076 ± 0.004	0.009 ± 0.000	0.368 ± 0.022
Red Grape	Yunnan, China	Peel	0.238 ± 0.008	0.065 ± 0.005	0.010 ± 0.001	0.313 ± 0.013
Rose Black Grape	Xinjiang, China	Peel	0.425 ± 0.010	0.212 ± 0.017	0.012 ± 0.001	0.649 ± 0.027
Rose Black Grape	Yunnan, China	Peel	0.326 ± 0.015	0.132 ± 0.006	0.010 ± 0.001	0.468 ± 0.022
Seedless Black Grape	California, CA, USA	Peel	0.326 ± 0.028	0.197 ± 0.004	0.012 ± 0.001	0.535 ± 0.033
Seedless Black Grape	Xinjiang, China	Peel	0.642 ± 0.031	0.331 ± 0.026	0.009 ± 0.000	0.982 ± 0.056
Seedless Dew Grape	Xinjiang, China	Peel	0.163 ± 0.003	0.092 ± 0.003	0.012 ± 0.001	0.266 ± 0.007
Seedless Green Grape	Xinjiang, China	Peel	0.126 ± 0.009	0.061 ± 0.000	0.012 ± 0.001	0.198 ± 0.010
Seedless Red Grape	California, CA, USA	Peel	0.199 ± 0.005	0.075 ± 0.002	0.017 ± 0.000	0.291 ± 0.006
Seedless Red Grape	Victoria, Australia	Peel	0.226 ± 0.010	0.091 ± 0.003	0.008 ± 0.000	0.325 ± 0.013
Seedless Red Grape	Xinjiang, China	Peel	0.321 ± 0.013	0.106 ± 0.000	0.008 ± 0.001	0.435 ± 0.014
Seedless Red Grape	Yunnan, China	Peel	0.317 ± 0.001	0.125 ± 0.010	0.009 ± 0.000	0.451 ± 0.011
Summer Black Grape	Shaanxi, China	Peel	0.317 ± 0.015	0.111 ± 0.008	0.014 ± 0.001	0.441 ± 0.023
Summer Black Grape	Xinjiang, China	Peel	0.403 ± 0.010	0.196 ± 0.015	0.011 ± 0.001	0.609 ± 0.025
Black Grape	Yunnan, China	Seed	1.126 ± 0.044	0.173 ± 0.003	0.041 ± 0.001	1.339 ± 0.048
Ito Kyoho Grape	Yunnan, China	Seed	2.989 ± 0.017	0.109 ± 0.005	0.024 ± 0.000	3.122 ± 0.022
Kyoho Grape	Xinjiang, China	Seed	3.786 ± 0.182	0.078 ± 0.006	0.020 ± 0.001	3.884 ± 0.189
Kyoho Grape	Guangxi, China	Seed	2.636 ± 0.238	0.096 ± 0.004	0.033 ± 0.002	2.765 ± 0.245
Kyoho Grape	Yunnan, China	Seed	1.165 ± 0.051	0.074 ± 0.006	0.023 ± 0.002	1.262 ± 0.059
Pearl Black Grape	Xinjiang, China	Seed	3.378 ± 0.167	0.101 ± 0.002	0.146 ± 0.006	3.626 ± 0.176
Red Grape	Yunnan, China	Seed	3.792 ± 0.211	0.126 ± 0.001	0.038 ± 0.001	3.957 ± 0.213
Red Grape	Guangxi, China	Seed	1.165 ± 0.022	0.157 ± 0.010	0.040 ± 0.002	1.361 ± 0.033
Red Grape	Xinjiang, China	Seed	2.536 ± 0.227	0.114 ± 0.000	0.030 ± 0.001	2.680 ± 0.227
Red Grape	California, CA, USA	Seed	1.024 ± 0.044	0.086 ± 0.008	0.020 ± 0.002	1.130 ± 0.054

**Table 5:** Phenolic components of peels and seeds from 30 grape varieties.

Name of Grapes	Place of Production	Part of Grapes	Phenols	Total Contents (mg/g FW)
Black Grape	Yunnan, China	Peel	cyanidin-3-glucoside	0.174 ± 0.009
			ferulaic acid	0.241 ± 0.011
			rutin	0.073 ± 0.006
			resveratrol	0.266 ± 0.015
Blackcurrant Grape	California, CA, USA	Peel	cyanidin-3-glucoside	0.498 ± 0.028
			rutin	0.687 ± 0.047
Flame Grape	Xinjiang, China	Peel	cyanidin-3-glucoside	0.421 ± 0.023
			ferulaic acid	0.049 ± 0.003
			rutin	0.367 ± 0.015
Fragrant Green Grape	Yunnan, China	Peel	rutin	0.383 ± 0.019
Golden Finger Grape	California, CA, USA	Peel	cyanidin-3-glucoside	0.150 ± 0.007
			ferulaic acid	0.041 ± 0.003
			rutin	0.569 ± 0.034
Green Grape	Victoria, Australia	Peel	rutin	0.268 ± 0.025
Ito Kyoho Grape	Yunnan, China	Peel	epicatechin	0.015 ± 0.001
			rutin	0.035 ± 0.003
Kyoho Grape	Liaoning, China	Peel	rutin	0.113 ± 0.005
Kyoho Grape	Xinjiang, China	Peel	epicatechin	0.026 ± 0.002
			rutin	0.129 ± 0.009
Kyoho Grape	Guangxi, China	Peel	rutin	0.138 ± 0.008
Kyoho Grape	Yunnan, China	Peel	rutin	0.117 ± 0.006
Pearl Black Grape	Xinjiang, China	Peel	rutin	0.199 ± 0.007
Pearl Green Grape	Xinjiang, China	Peel	rutin	0.016 ± 0.000
Pearl Green Grape	Victoria, Australia	Peel	rutin	0.047 ± 0.002
Red Grape	Yunnan, China	Peel	cyanidin-3-glucoside	0.326 ± 0.023
			rutin	0.804 ± 0.055
Red Grape	Guangxi, China	Peel	cyanidin-3-glucoside	0.211 ± 0.007
			rutin	0.293 ± 0.026
Red Grape	Xinjiang, China	Peel	cyanidin-3-glucoside	0.412 ± 0.033
			rutin	0.298 ± 0.027
Red Grape	California, China	Peel	cyanidin-3-glucoside	0.377 ± 0.030
			rutin	0.298 ± 0.020
Rose Black Grape	Xinjiang, China	Peel	rutin	0.137 ± 0.006
Rose Black Grape	Yunnan, China	Peel	rutin	0.030 ± 0.001
Seedless Black Grape	Xinjiang, China	Peel	rutin	0.059 ± 0.002
Seedless Black Grape	California, CA, USA	Peel	rutin	0.265 ± 0.022
Seedless Dew Grape	Xinjiang, China	Peel	rutin	0.049 ± 0.001
Seedless Green Grape	Xinjiang, China	Peel	rutin	0.008 ± 0.000
Seedless Red Grape	Yunnan, China	Peel	rutin	0.176 ± 0.012
Seedless Red Grape	Xinjiang, China	Peel	cyanidin-3-glucoside	0.021 ± 0.001
			rutin	0.195 ± 0.013
Seedless Red Grape	California, CA, USA	Peel	cyanidin-3-glucoside	0.058 ± 0.003
			rutin	0.666 ± 0.056
Seedless Red Grape	Victoria, Australia	Peel	cyanidin-3-glucoside	0.272 ± 0.011
			rutin	0.594 ± 0.036
Summer Black Grape	Shaanxi, China	Peel	epicatechin	0.051 ± 0.004
			rutin	0.150 ± 0.006
Summer Black Grape	Xinjiang, China	Peel	rutin	0.125 ± 0.003
Black Grape	Yunnan, China	Seed	gallic acid	0.146 ± 0.008
			cyanidin-3-glucoside	0.305 ± 0.028
			epicatechin	1.207 ± 0.074
			catechin gallate	0.052 ± 0.002
Ito Kyoho Grape	Yunnan, China	Seed	gallic acid	0.054 ± 0.003
			cyanidin-3-glucoside	0.840 ± 0.052
			epicatechin	1.693 ± 0.094
			catechin gallate	0.028 ± 0.002
Kyoho Grape	Xinjiang, China	Seed	gallic acid	0.066 ± 0.003
			cyanidin-3-glucoside	0.180 ± 0.011
			epicatechin	2.088 ± 0.106
			catechin gallate	0.119 ± 0.004
Kyoho Grape	Guangxi, China	Seed	gallic acid	0.052 ± 0.002
			cyanidin-3-glucoside	0.105 ± 0.005
			epicatechin	2.039 ± 0.187
			catechin gallate	0.044 ± 0.002
Kyoho Grape	Yunnan, China	Seed	gallic acid	0.087 ± 0.002
			cyanidin-3-glucoside	0.202 ± 0.019
			epicatechin	1.886 ± 0.165
			catechin gallate	0.054 ± 0.002
Pearl Black Grape	Xinjiang, China	Seed	gallic acid	0.193 ± 0.017
			cyanidin-3-glucoside	0.189 ± 0.009
			epicatechin	1.745 ± 0.111
			catechin gallate	0.126 ± 0.005
Red Grape	Yunnan, China	Seed	gallic acid	0.236 ± 0.009
			cyanidin-3-glucoside	0.113 ± 0.003
			epicatechin	2.156 ± 0.156
			catechin gallate	0.176 ± 0.008
Red Grape	Guangxi, China	Seed	gallic acid	0.089 ± 0.004
			cyanidin-3-glucoside	0.095 ± 0.005
			epicatechin	1.547 ± 0.144
			catechin gallate	0.145 ± 0.005
Red Grape	Xinjiang, China	Seed	gallic acid	0.056 ± 0.002
			cyanidin-3-glucoside	0.058 ± 0.003
			epicatechin	1.644 ± 0.098
			catechin gallate	0.128 ± 0.004
Red Grape	California, CA, USA	Seed	gallic acid	0.022 ± 0.001
			cyanidin-3-glucoside	0.111 ± 0.005
			epicatechin	0.877 ± 0.065
			catechin gallate	0.165 ± 0.013

**Table 6:** Moisture contents of the tested grape peels and seeds.

Name of Grapes	Place of Production	Part of Grapes	Moisture Contents (%)
Black Grape	Yunnan, China	Peel	72.333 ± 2.951
Blackcurrant Grape	California, CA, USA	Peel	72.382 ± 1.023
Flame Grape	Xinjiang, China	Peel	58.699 ± 2.487
Fragrant Green Grape	Yunnan, China	Peel	74.926 ± 3.156
Golden Finger Grape	California, CA, USA	Peel	71.851 ± 2.894
Green Grape	Victoria, Australia	Peel	79.027 ± 0.525
Ito Kyoho Grape	Yunnan, China	Peel	77.103 ± 3.446
Kyoho Grape	Guangxi, China	Peel	77.402 ± 1.568
Kyoho Grape	Liaoning, China	Peel	80.564 ± 3.699
Kyoho Grape	Xinjiang, China	Peel	78.757 ± 3.321
Kyoho Grape	Yunnan, China	Peel	79.920 ± 3.219
Pearl Black Grape	Xinjiang, China	Peel	79.023 ± 3.013
Pearl Green Grape	Xinjiang, China	Peel	74.881 ± 0.856
Pearl Green Grape	Victoria, Australia	Peel	75.233 ± 3.112
Red Grape	California, CA, USA	Peel	76.320 ± 3.239
Red Grape	Guangxi, China	Peel	77.416 ± 2.986
Red Grape	Xinjiang, China	Peel	73.806 ± 2.357
Red Grape	Yunnan, China	Peel	76.117 ± 2.766
Rose Black Grape	Xinjiang, China	Peel	69.404 ± 2.551
Rose Black Grape	Yunnan, China	Peel	69.726 ± 0.469
Seedless Black Grape	California, CA, USA	Peel	72.423 ± 2.995
Seedless Black Grape	Xinjiang, China	Peel	69.200 ± 2.210
Seedless Dew Grape	Xinjiang, China	Peel	74.168 ± 2.848
Seedless Green Grape	Xinjiang, China	Peel	72.232 ± 2.365
Seedless Red Grape	California, CA, USA	Peel	71.103 ± 1.334
Seedless Red Grape	Victoria, Australia	Peel	66.384 ± 2.085
Seedless Red Grape	Xinjiang, China	Peel	73.000 ± 2.462
Seedless Red Grape	Yunnan, China	Peel	73.293 ± 2.366
Summer Black Grape	Shaanxi, China	Peel	71.560 ± 2.232
Summer Black Grape	Xinjiang, China	Peel	69.450 ± 2.211
Black Grape	Yunnan, China	Seed	42.396 ± 1.845
Ito Kyoho Grape	Yunnan, China	Seed	44.002 ± 1.933
Kyoho Grape	Xinjiang, China	Seed	41.664 ± 0.759
Kyoho Grape	Guangxi, China	Seed	43.489 ± 1.926
Kyoho Grape	Yunnan, China	Seed	44.001 ± 1.988
Pearl Black Grape	Xinjiang, China	Seed	46.646 ± 2.003
Red Grape	Yunnan, China	Seed	52.859 ± 2.109
Red Grape	Guangxi, China	Seed	51.622 ± 0.221
Red Grape	Xinjiang, China	Seed	46.424 ± 1.903
Red Grape	California, CA, USA	Seed	51.570 ± 2.158
